# Supplementary figures and images for: ATF3 downmodulates its new targets IFI6 and IFI27 to suppress the growth and migration of tongue squamous cell carcinoma cells
Source: PLoS Genet. 2021 Feb 4;17(2):e1009283. doi: 10.1371/journal.pgen.1009283 (PMC7888615; doi:10.1371/journal.pgen.1009283)

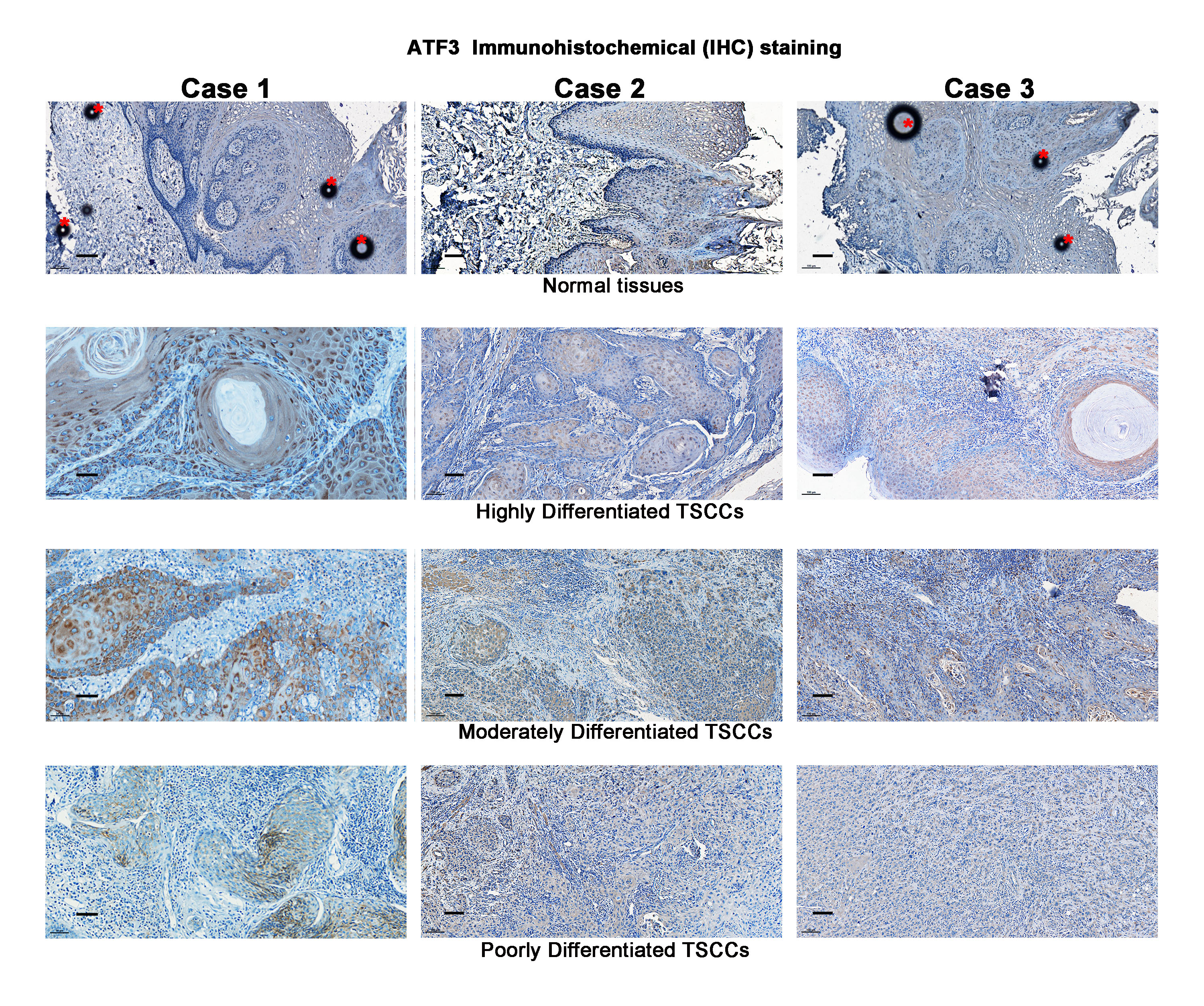

Supplement: S1 Fig — Immunochemical (IHC) staining of ATF3 was performed with different grade clinical TSCC tissues from Fig 1A, and representative staining images of 3 patients (cases 1, 2 and 3) from each group as indicated are shown. * indicates artificial bubbles. A high magnification image of Case 1 from each group is shown in Fig 1B. Scale bars = 100 μm. (JPG) [file pgen.1009283.s001.jpg]

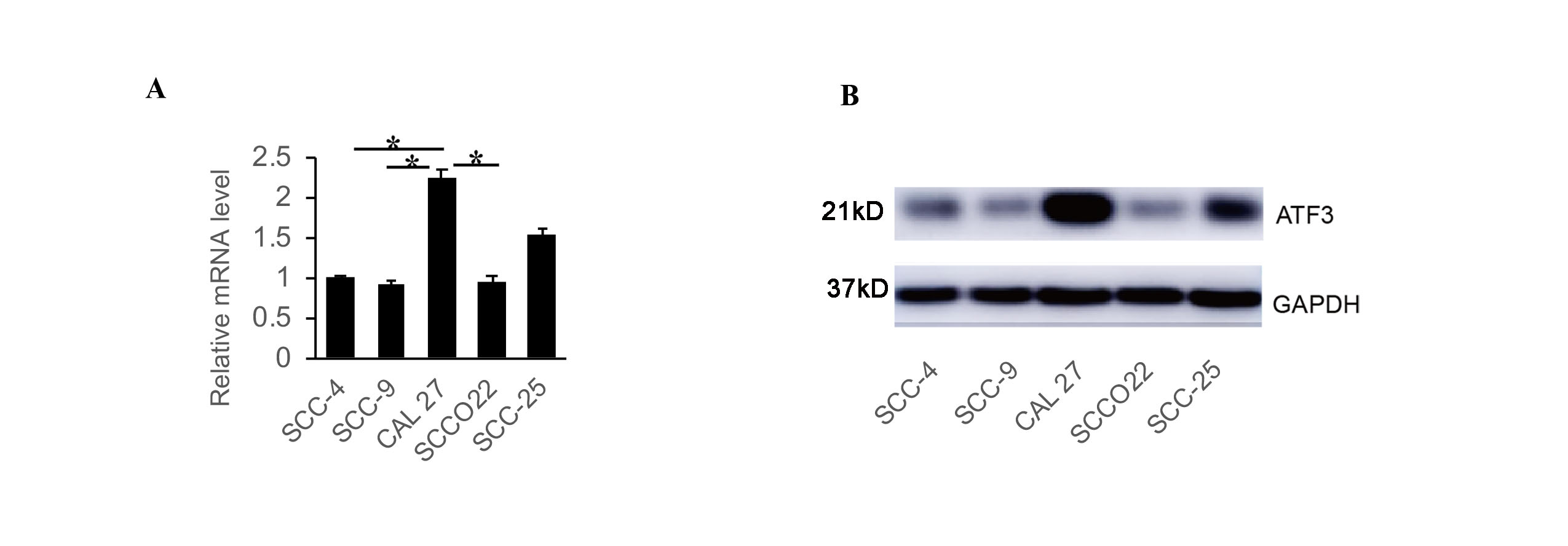

Supplement: S2 Fig — A, B. ATF3 expression levels in different TSCC cell lines were analyzed by RT-PCR (A) and by western blot (B). *p<0.05 when comparing the two groups as indicated. (JPG) [file pgen.1009283.s002.jpg]

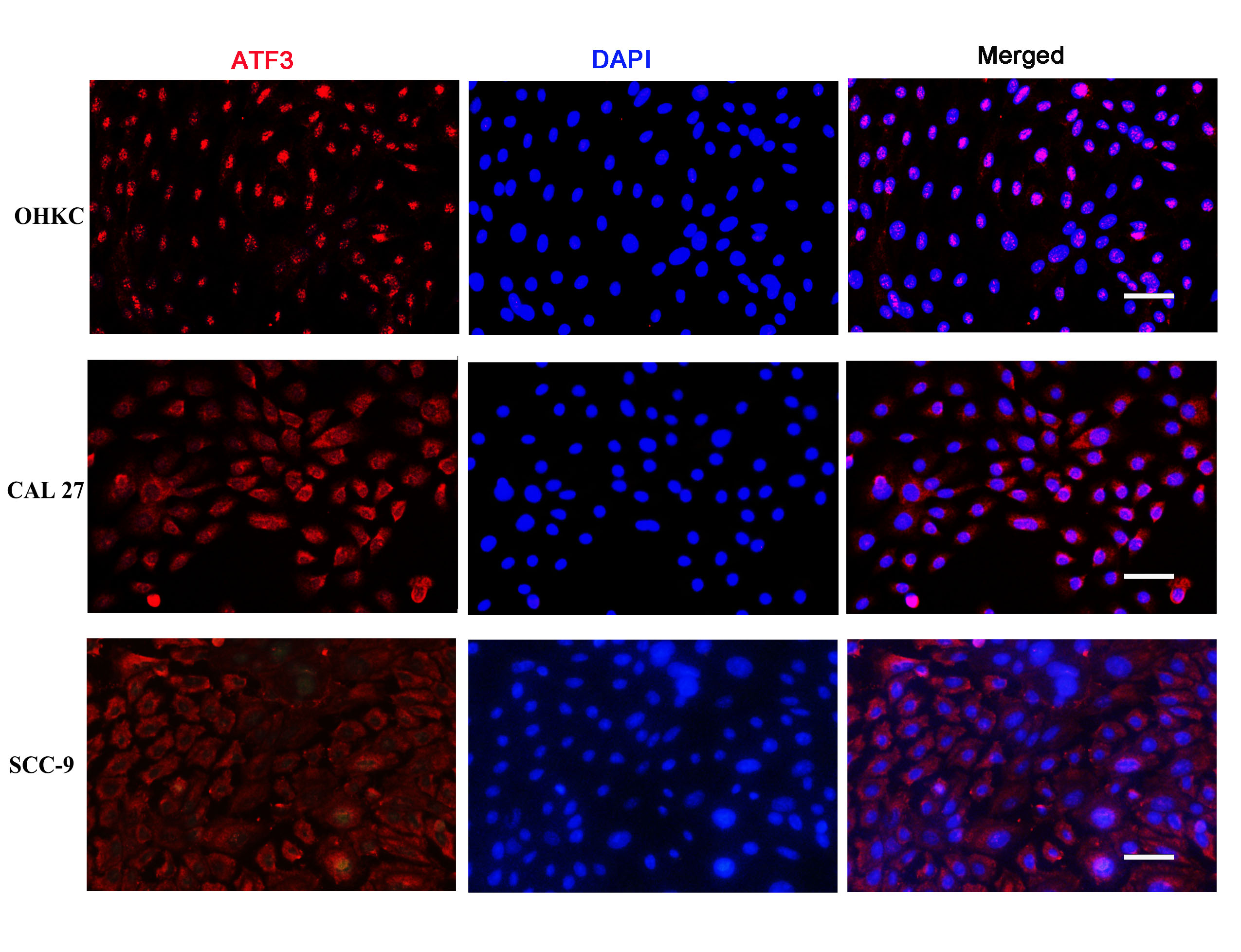

Supplement: S3 Fig — Representative images of immunofluorescence staining of ATF3 (red, left panels) and DAPI (blue, middle panels) in cultured primary oral human keratinocytes (OHKCs), CAL 27 and SCC-9 cells. Merged images of ATF3 and DAPI staining are shown in the right panels, which are also shown in Fig 1C. DAPI for nuclei staining. Scale bars = 100 μm. (JPG) [file pgen.1009283.s003.jpg]

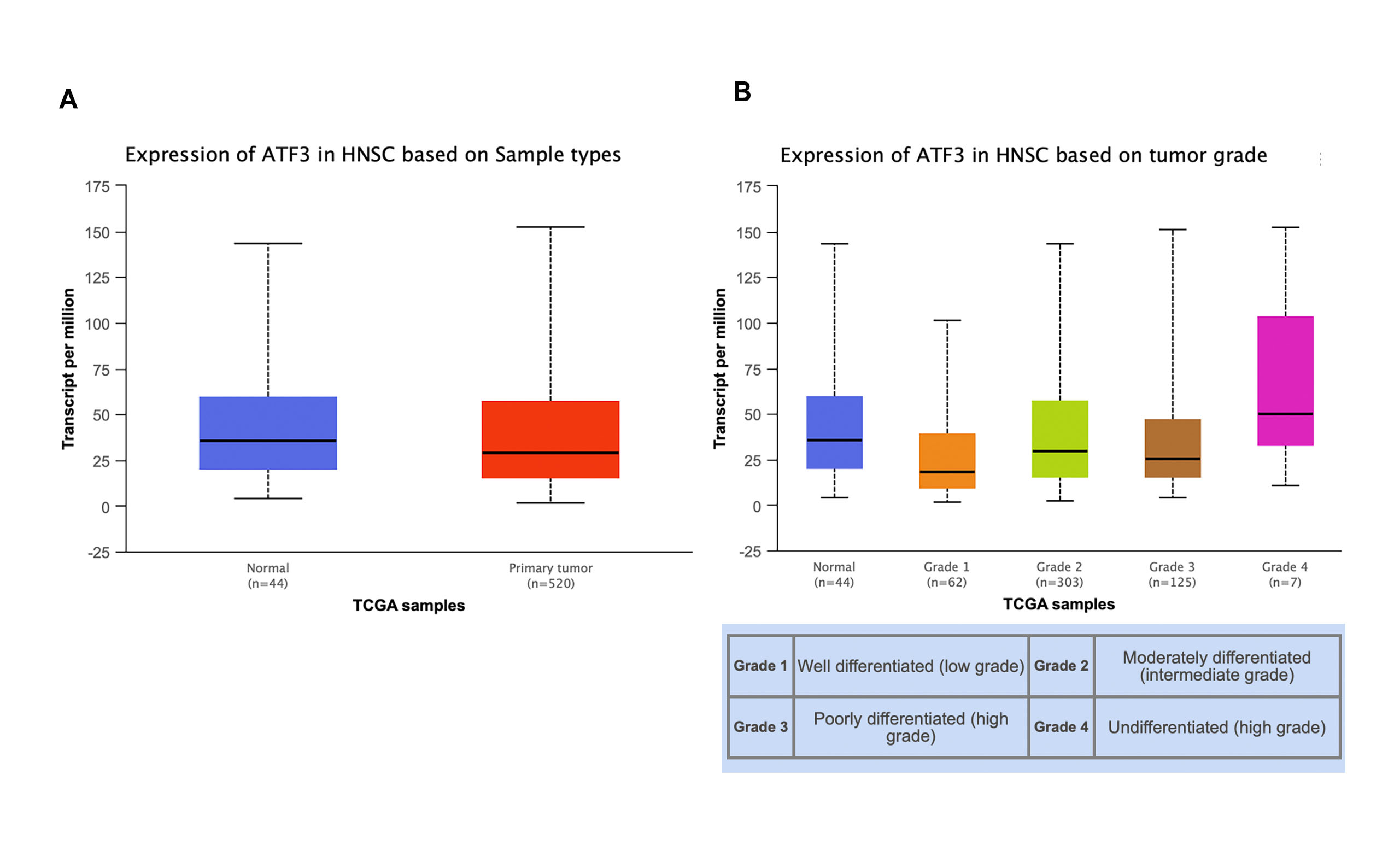

Supplement: S4 Fig — Data analysis in A was performed at the following link: http://ualcan.path.uab.edu/cgi-bin/TCGAExResultNew2.pl?genenam=ATF3&ctype=HNSC; Data analysis in B was performed at the following link: http://ualcan.path.uab.edu/cgi-bin/TCGAExResultNew2.pl?genenam=ATF3&ctype=HNSC (JPG) [file pgen.1009283.s004.jpg]

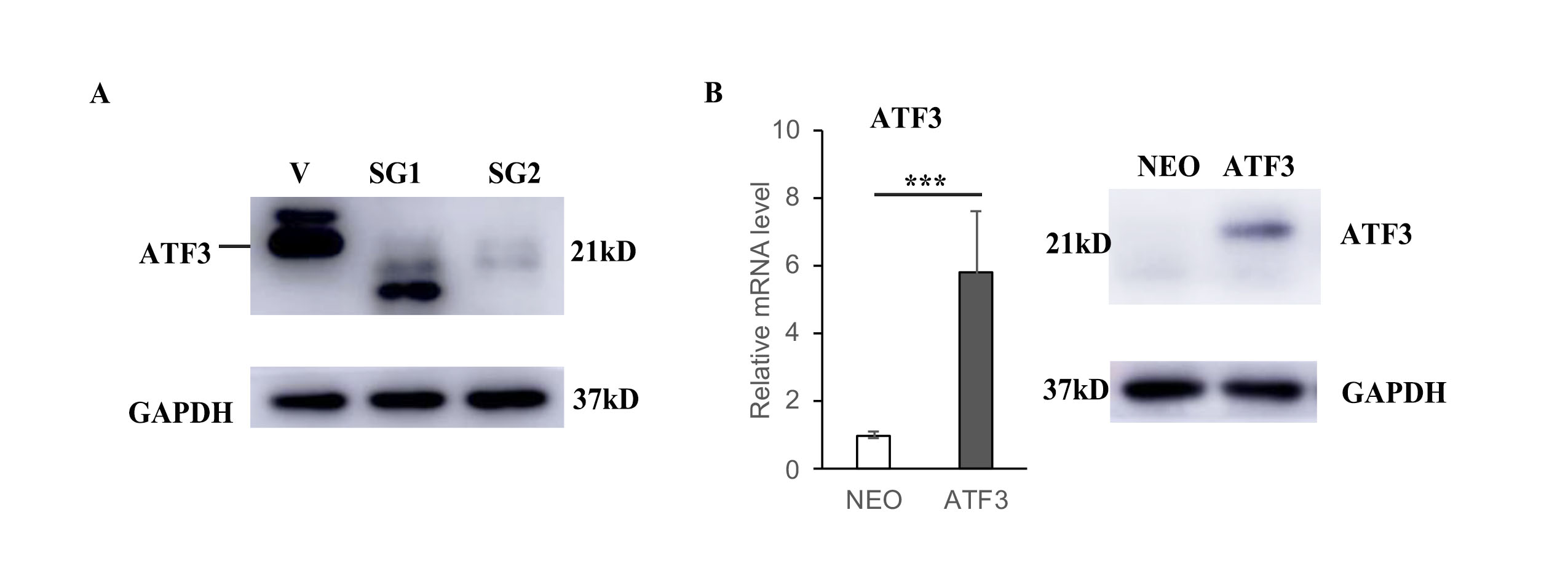

Supplement: S5 Fig — A. CAL 27 cells infected with a lentivirus carrying CRISPR/Cas9 with ATF3 sgRNA1 (SG1) or sgRNA2 (SG2) or with an empty vector as a control (V). After selection with puromycin, the cells were collected for western blot analysis of ATF3 protein to determine the deletion efficiency. GAPDH was used as a housekeeping gene for a loading control. B. SCC-9 cells were infected with a retrovirus expressing ATF3 (ATF3) or neomycin (NEO) as a control; 48 h after infection, the cells were collected for RT-PCR (left panel) and western blot analysis for ATF3 expression. Relative mRNA levels of ATF3 were normalized with the 36beta4 gene, and GAPDH was used as a loading control for the protein level. ***p<0.005 compared with the control group. (JPG) [file pgen.1009283.s005.jpg]

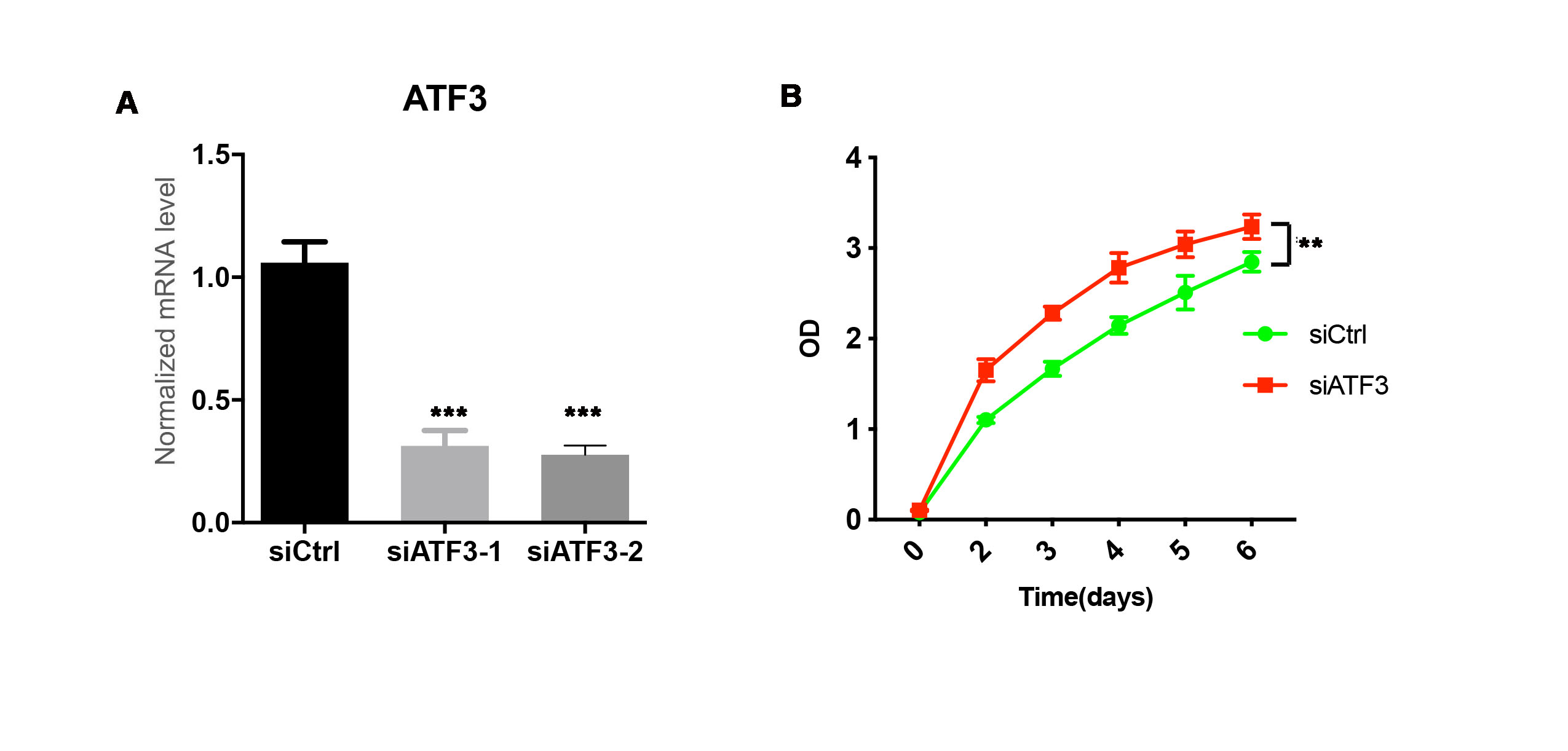

Supplement: S6 Fig — A. CAL 27 cells were transfected with two independent siRNAs of ATF3 or with a scrambled siRNA (siCtrl). 72 h after transfection, the cells were collected for RT-PCR analysis for ATF3 expression. The relative mRNA levels of IFI6 and IFI27 were normalized with the 36beta4 gene, ***p<0.005 compared with the control group (siCtrl) as indicated. B. Growth of CAL 27 cells transfected with siRNAs of ATF3 or with a scrambled siRNA (siCtrl) were analyzed using a CCK8 kit at different time points. **p<0.01 compared with the control siCtrl group. (JPG) [file pgen.1009283.s006.jpg]

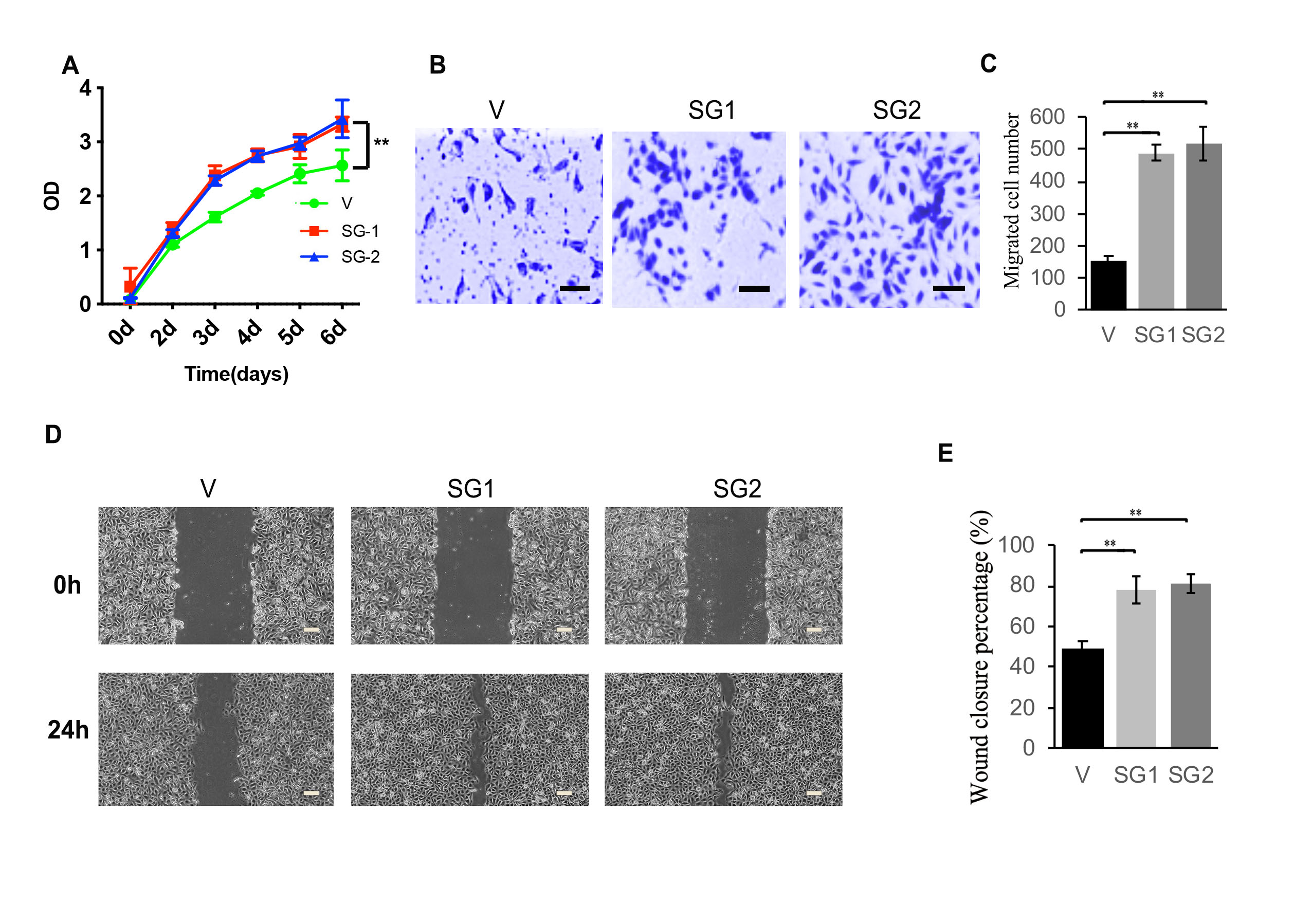

Supplement: S7 Fig — A. Growth of SCC-25 cells with the CRISPR/Cas9 mediated deletion of ATF3 (SG1 or SG2) or with the empty vector as a control (V) were analyzed using a CCK8 kit at different time points. **p<0.01 when compared with the control group. B, C. Trans-well migration assays were performed with ATF3-deleted (SG1 or SG2) or control (V) TSCC cells; images of migrated cells at 24 h are shown in B, and the numbers of migrated cells in the different groups are shown in C. **p<0.01 compared with the control group. Scale bars in B = 100 μm. D, E. Wound-healing assays were performed with ATF3-deleted (SG1 or SG2) or control (V) TSCC cells, representative wound healing images at 0 h and 24 h after wounding are shown in D, and the percentage of wound closure is calculated in E. ** p<0.01 when compared with the control group. Scale bars in D = 100 μm. (JPG) [file pgen.1009283.s007.jpg]

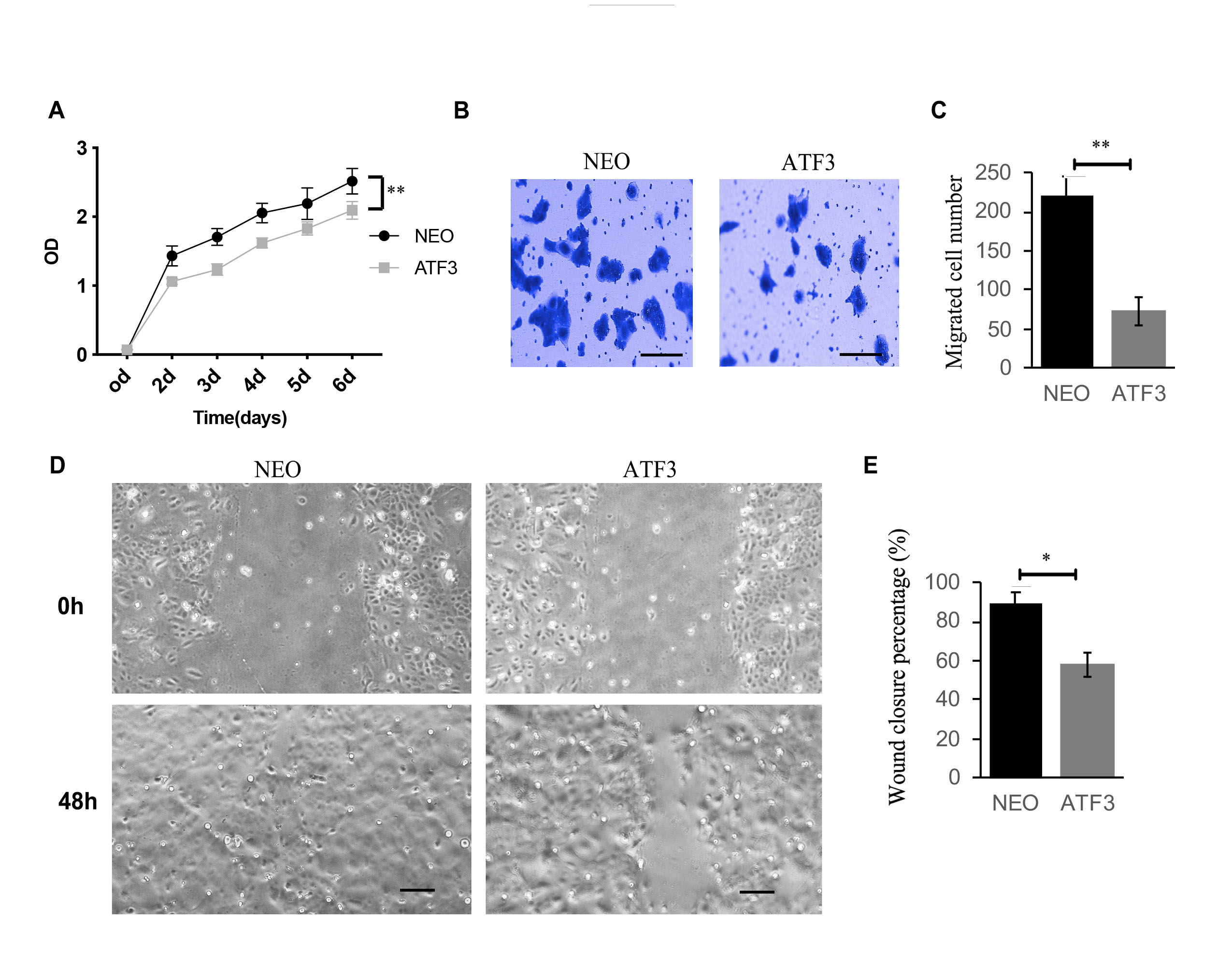

Supplement: S8 Fig — A. Growth of SCC-4 TSCC cells infected with a retrovirus overexpressing ATF3 (ATF3) or expressing neomycin as a control (NEO) were analyzed using a CCK8 kit at different time points. **p<0.01 compared with the control group. B, C. Trans-well migration assays were performed with TSCC cells overexpressing ATF3 (ATF3) or control NEO; images of migrated cells at 24 h are shown in B, and the numbers of cells that migrated through the filter in the different groups are shown in C. **p<0.01 compared with the control. D, E. Wound-healing assays were performed with TSCC cells overexpressing ATF3 (ATF3) or control NEO (NEO); representative images at 0 h and 48 h after wounding are shown in D, and the percentage of wound closure at 48 h after wounding is calculated in E. *p<0.05 compared with the control group as indicated. Scale bars in B and D = 200 μm. (JPG) [file pgen.1009283.s008.jpg]

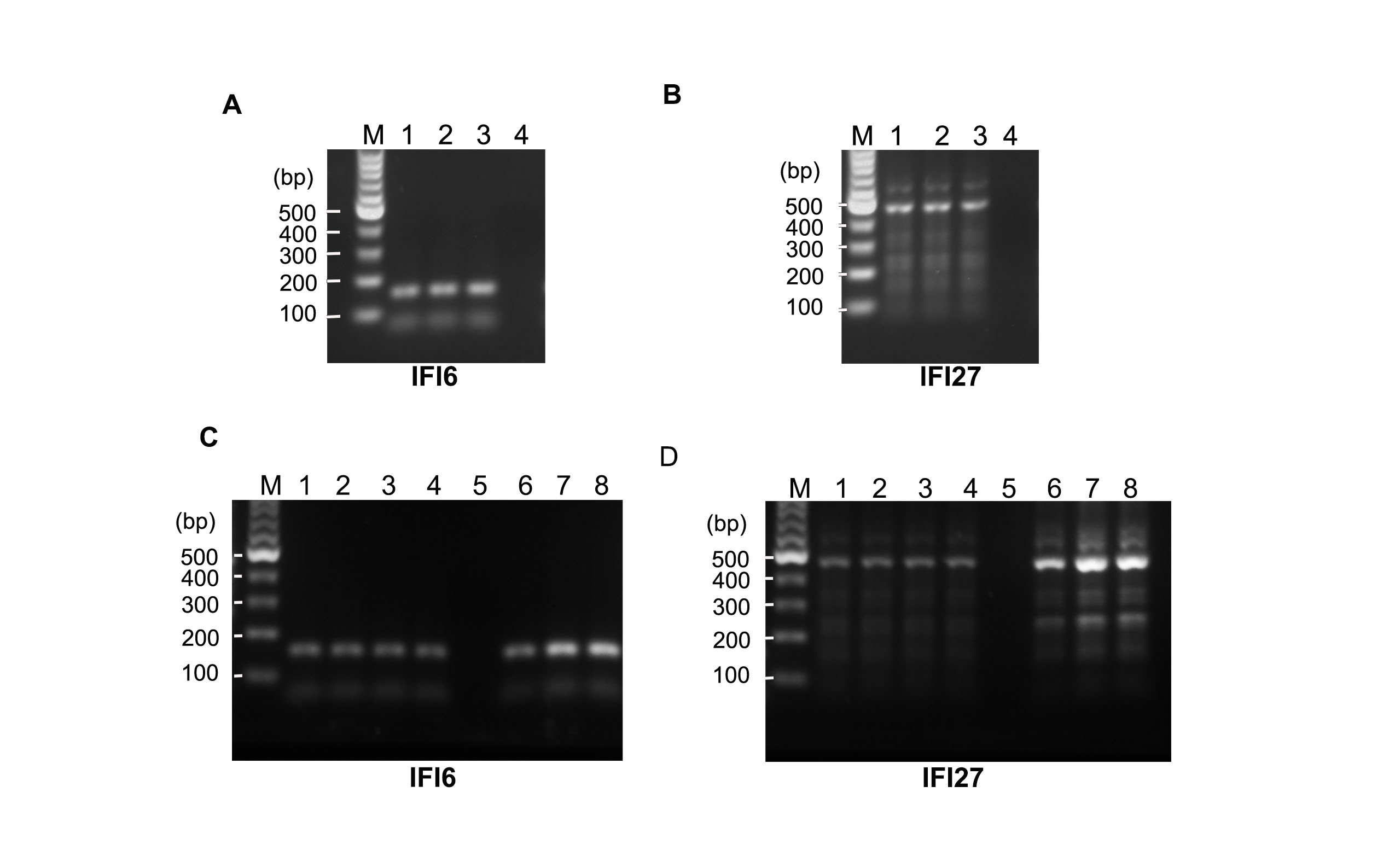

Supplement: S9 Fig — A,B. Extracts of CAL 27 cells were processed for CHIP assays with the anti-ATF3 antibody and non-immune IgG followed by PCR analysis of either the IFI6 (A) or the IFI27 promoter regions containing ATF3 binding sites as shown (maps) in Fig 4E and 4F. Gel electrophoresis (1% agarose gel) shows the PCR amplified 160 bp fragment for IFI6 and the 463 bp fragment for IFI27. Labels for lanes: M: molecular markers, 1: Input, 2,3: ATF3 antibody; 4: IgG. C,D. Extracts of CAL 27 cells with either the deletion of ATF3 (SG1 or SG2) or the overexpression of ATF3 (ATF3) and the corresponding controls (V or NEO) were processed for CHIP assays with the anti-ATF3 antibody or non-immune IgG followed by PCR analysis of either IFI6 (C) or IFI27 (D) promoter regions containing ATF3 binding sites. PCR products were analyzed by gel electrophoresis. Labels for lanes: M: molecular markers, 1: Input, 2: V+ATF3 antibody, 3: SG1+ATF3 antibody, 4: SG2+ATF3 antibody; 5: IgG, 6: NEO+ATF3 antibody, 7–8: overexpression of ATF3+ATF3 antibody. (JPG) [file pgen.1009283.s009.jpg]

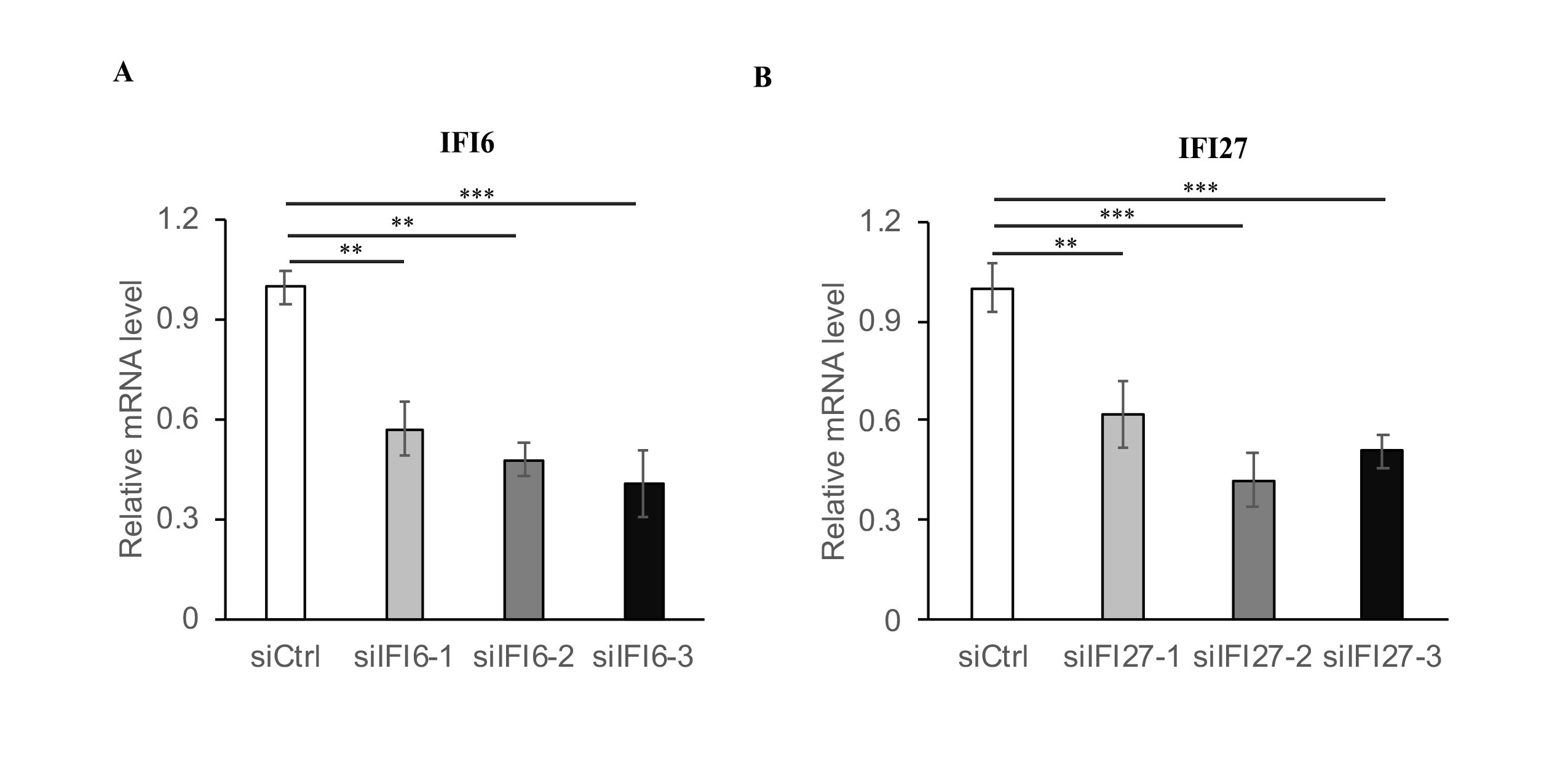

Supplement: S10 Fig — A, B. CAL 27 cells were transfected with 3 independent siRNAs of IFI6 (A) or IFI27 (B) or a scrambled siRNA (siCtrl). 72 h after transfection, the cells were collected for RT-PCR analysis of IFI6 and IFI27 expression. The relative mRNA levels of IFI6 or IFI27 were normalized with the 36beta4 gene, **p<0.01, ***p<0.005 compared with the control group (siCtrl) as indicated. (JPG) [file pgen.1009283.s010.jpg]

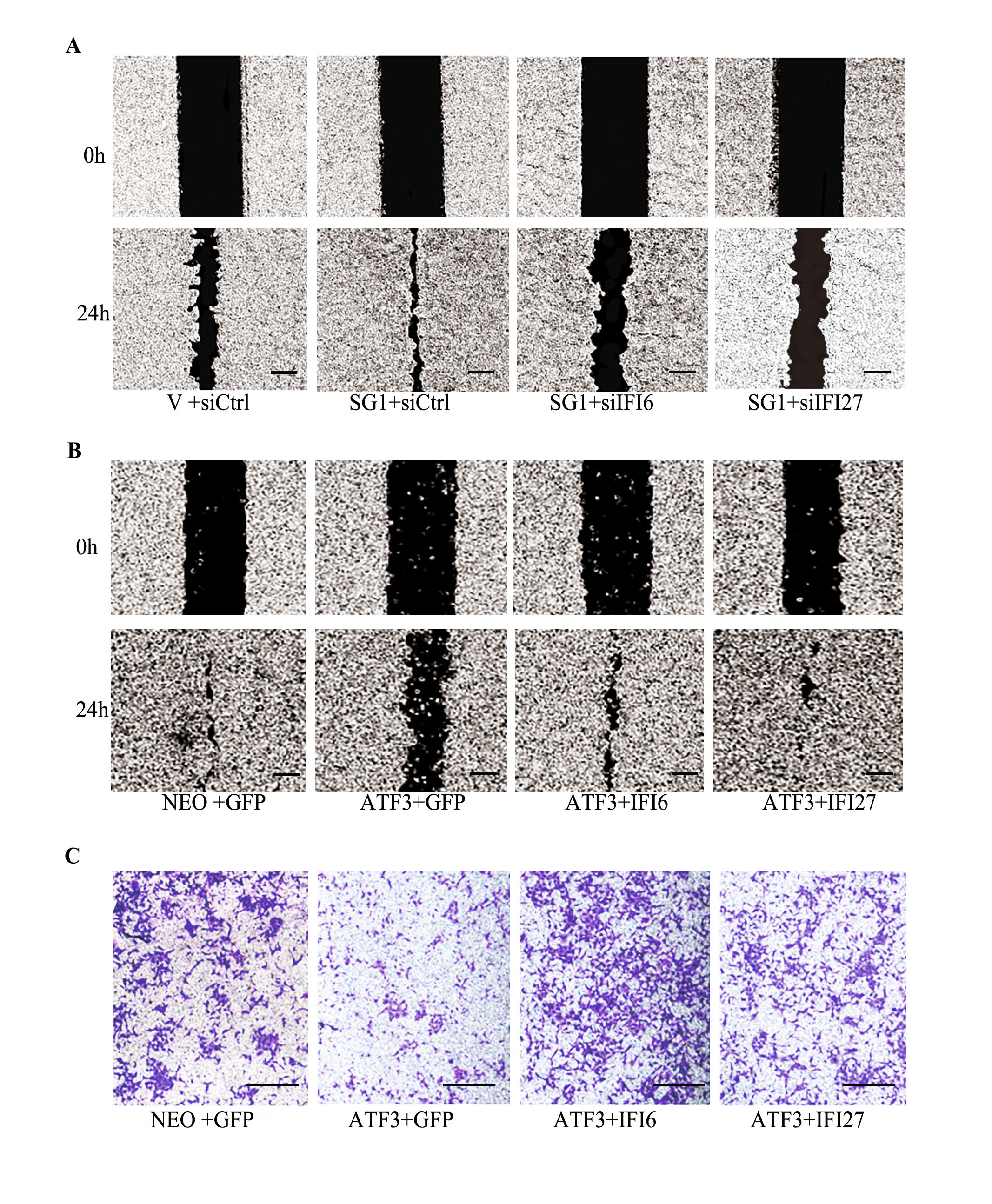

Supplement: S11 Fig — A, Wound healing migration assays were performed with CAL 27 cells in the same groups as in Fig 5A. Images of wound healing at 24 h after wounding are shown here and quantification of the wound healing percentage is shown in Fig 5E. B. Wound healing migration assays were performed with SCC-9 cells in the same groups as in Fig 5C. Images of wound healing at 24 h after wounding are shown here and quantification of the wound healing percentage is shown in Fig 5F. C. Trans-well migration assays were performed with cells in the same groups as in Fig 5C. Representative images of migrated cells at 24 h after wounding are shown here, and the numbers of migrated cells in the different groups are shown in Fig 5G. Scale bars = 200 μm. (JPG) [file pgen.1009283.s011.jpg]

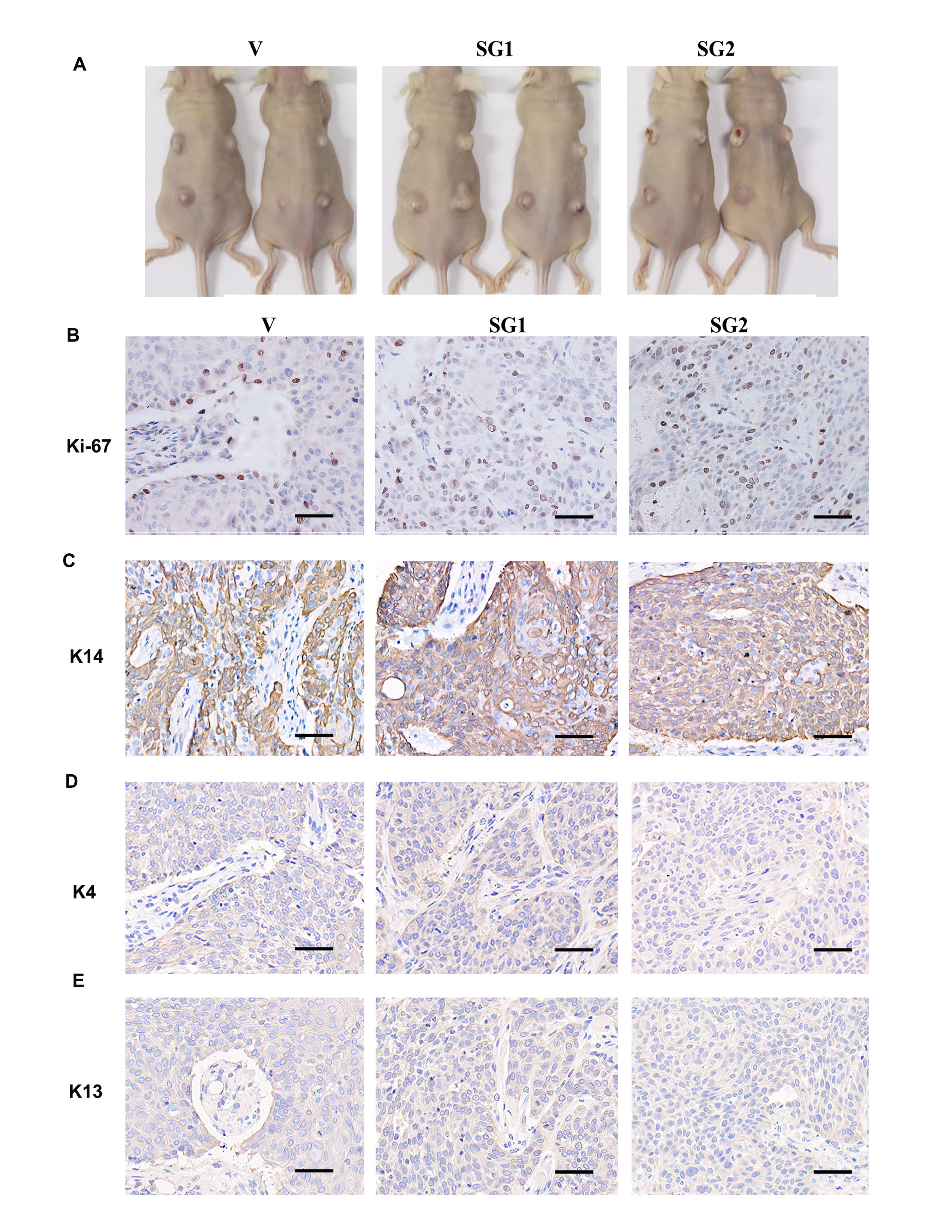

Supplement: S12 Fig — A. Representative images of mice with TSCC tumors formed from grafts of CAL 27 cells with the deletion of ATF3 (SG1 or SG2) or the control group (V). B-E. Representative images of IHC staining for Ki-67 (B), K14 (C), K4 (D) and K13 (E) in tumors from the indicated groups as shown in A. High magnification images of Ki-67 and K14 staining are also shown in Fig 6C. Scale bars = 100 μm. (JPG) [file pgen.1009283.s012.jpg]

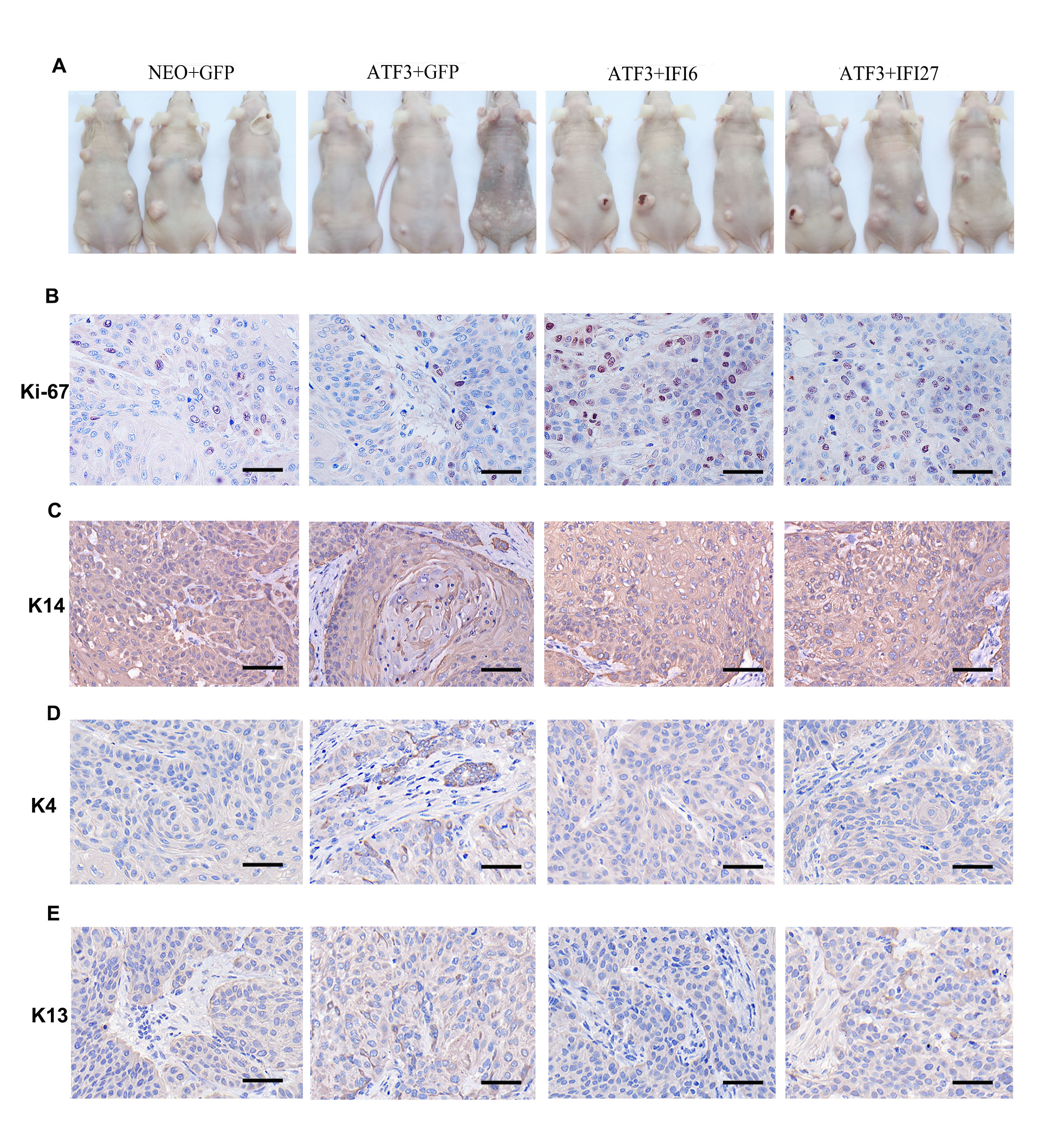

Supplement: S13 Fig — A. Representative images of mice with tumors formed from grafts of SCC-9 cells with overexpression of ATF3 (ATF3) plus overexpression of IFI6 (ATF3+IFI6) or IFI27 (ATF3+IFI27), and NEO as a control group. B-E. Representative images of IHC staining of Ki-67 (B), K14 (C), K4 (D) and K13 (E) in tumors from the indicated groups as shown in A. High magnification images of these staining patterns are also shown in Fig 6G, 6I, 6K and 6M. Scale bars = 100 μm. (JPG) [file pgen.1009283.s013.jpg]
