# Supplementary material for: ATF3 downmodulates its new targets IFI6 and IFI27 to suppress the growth and migration of tongue squamous cell carcinoma cells
Source: PLoS Genet. 2021 Feb 4;17(2):e1009283. doi: 10.1371/journal.pgen.1009283 (PMC7888615; doi:10.1371/journal.pgen.1009283)
Supplement: S1 Table — (PDF) [file pgen.1009283.s014.pdf]

S1 Table. Oligo sequences used for RT-PCR analysis

Gene	Forward Primers	Reverse Primers
ATF3	GTCCATCACAAAAGCCGAGG	GCACTCCGTCTTCTCCTTCT
IFI6	GATGAGCTGGTCTGCGATCC	TCGAGATACTTGTGGGTGGC
IFI27	CCTTCTTTGGGTCTGGCTGA	CCACACTGGTCACTGCTGAT
H36beta4	GCAATGTTGCCAGTGTCTGT	GCCTTGACCTTTTCAGCAAG
