# Supplementary material for: ATF3 downmodulates its new targets IFI6 and IFI27 to suppress the growth and migration of tongue squamous cell carcinoma cells
Source: PLoS Genet. 2021 Feb 4;17(2):e1009283. doi: 10.1371/journal.pgen.1009283 (PMC7888615; doi:10.1371/journal.pgen.1009283)
Supplement: S2 Table — (PDF) [file pgen.1009283.s015.pdf]

S2 Table. Oligo sequences used for CHIP assays

Gene	Forward Primers	Reverse Primers
IFI6	GCATCACCATCCAAAGGCTT	ATTGTTCTCTCAGGCTGGCT
IFI27	CACTTGAATCACTCCACCGG	CAGCTTATCACATGGGCCAC
