# Supplementary material for: ATF3 downmodulates its new targets IFI6 and IFI27 to suppress the growth and migration of tongue squamous cell carcinoma cells
Source: PLoS Genet. 2021 Feb 4;17(2):e1009283. doi: 10.1371/journal.pgen.1009283 (PMC7888615; doi:10.1371/journal.pgen.1009283)
Supplement: S3 Table — (PDF) [file pgen.1009283.s016.pdf]

S3 Table. Oligo sequences used for siRNAs

IFI6	siRNA1	sense	5'- GCAGCGUCGUCAUAGGUAATT -3'
		antisense	5'- UUACCUAUGACGACGCUGCTT-3'
	siRNA2	sense	5'- CCACAAGUAUCUCGAUAGUTT -3'
		antisense	5'- ACUAUCGAGAUACUUGUGGTT-3'
	siRNA3	sense	5'- GCUGCUCUUCACUUGCAGUTT-3'
		antisense	5'- ACUGCAAGUGAAGAGCAGCTT -3'
IFI27	siRNA1	sense	5'- GGCCAGGAUUGCUACAGUUTT-3'
		antisense	5'- AACUGUAGCAAUCCUGGCCTT-3'
	siRNA2	sense	5'- GACUCUCCGGAUUGACCAATT-3'
		antisense	5'- UUGGUCAAUCCGGAGAGUCTT-3'
	siRNA3	sense	5'-GCUGUCAUUGCGAGGUUCUTT-3'
		antisense	5'- AGAACCUCGCAAUGACAGCTT-3'
ATF3	siRNA1	sense	5'-GGAAAGUGUGAAUGCUGAAdTdT-3’
		antisense	5’-UUCAGCAUUCACACUUUCCdTdT-3’
	siRNA2	sense	5’-CGUGCAGUAUCUCAAGAUAdTdT-3’
		antisense	5’-UAUCUUGAGAUACUGCACGdTdT-3’
