# Supplementary material for: ATF3 downmodulates its new targets IFI6 and IFI27 to suppress the growth and migration of tongue squamous cell carcinoma cells
Source: PLoS Genet. 2021 Feb 4;17(2):e1009283. doi: 10.1371/journal.pgen.1009283 (PMC7888615; doi:10.1371/journal.pgen.1009283)
Supplement: Attachment — Submitted filename: Comments-PGENETICS-D-20-00583.pdf [file pgen.1009283.s017.pdf]

Comments on PGENETICS-D-20-00583

SUMMARY

The study by Lin Xu and colleagues discovered a tumor suppressor role for activating transcription factor 3 ATF3 in tongue squamous cell carcinomas TSCCs through negative regulation of IFI6 and IFI27. This finding provides a potentially important angle to understand the TSCC etiology. A thorough understanding of ATF3 alteration and its downstream signaling pathways may pave the way for inventing new therapeutics against TSCCs. On a broad perspective, ATF3 regulation of IFI6 and IFI27 signaling could act as a fundamental mechanism conserved across diverse cancer forms and/or involved in normal development.

The authors have provided extensive molecular and cellular evidence, successfully connecting ATF3 function to the TSCC biology. Despite the strengths mentioned above, more experimental studies and conceptual clarification are required for general audience to efficiently and accurately digest the current manuscript.

1. Page 5: The authors state that "a potential redistribution of ATF3 from nuclei to the cytoplasm". This may not be true, since TSCC progression could theoretically prevent cytoplasmic ATF3 shuttling into the nucleus. The authors should revise the sentence – for example, ATF3 redistribution between nucleus and cytoplasm.

2. Suppl. Fig. 1: Is there any clinical staging difference between the five OSCC cell lines – highly, moderately, or poorly differentiated? Is there any scientific reason for using CAL27 and SCC-9 cells in this study?

3. Fig. 1C: Please add the individual images for ATF3 staining only.

4. Fig. 1E: Immunofluorescence quantification alone may not be enough to accurately determine the difference of nuclear/cytoplasmic ATF3 expression between the three cell lines. These observations should be confirmed by subcellular fractionation assay (cytoplasm/nucleoplasm/chromatin), followed by western blot analysis.

5. Fig. 1F: Please change the Y-axis values to a Log scale, to increase the readability.

6. Page 6: Please revise the conclusion paragraph, as no results directly support "the function of ATF3 is down-regulated", nor "disrupted localization of ATF3 from the nucleus to the cytoplasm".

7. Page 6 (the third from last line): Please change (Fig. 1E, F) to (Fig.1E), since Fig. 1F represents clinical data, rather than cell culture work.

8. Fig. 2F: Please repeat western blots in triplicate and perform relative quantification of both p-AKT/AKT and p-ERK/ERK in a bar chart (as illustrated in Fig. 3G).

9. Page 7: The authors state that "loss of ATF3 function promotes the malignant progression of oral SCCs". But, this seems to be an overstatement in the absence of direct evidence from transgenic animal studies tracing the steps of OSCC development *in vivo*.

10. Page 7 (the ninth from last line): Please change (Fig. 1F) to (Fig.1E).

11. Fig. 3B: Please replace the figure panel with color images (as shown in Fig. 2B).

12. Fig. 4B: Please add the SG2 data to RT-qPCR validation.

13. Fig. 4D: How many patient samples were subjected to IHC analysis? Please provide IHC scores for IFI6 and IFI27 in normal vs. TSCC specimens.

14. Fig. 4E: Please change the Y-axis values to a Log scale.

15. Figs. 5B and 5D: Which time point (day) was selected for RT-qPCR validation of knockdown/overexpression efficiency? This information should be added to the figure legend.

16. Fig. 5: There's no description/discussion on Figs. 5E, 5F, 5G, 5H, and 5I.

17. Fig. 6: If possible, please change Fig. 6 to a color figure.

18. Conceptual issue #1: It's unclear if the IFI6 and IFI27 genes are two direct targets of ATF3 (or its downstream effectors)? If yes, then ChIP-qPCR should be applied to prove ATF3 binding to the IFI6 and IFI27 promoters. If no, then "ATF3 downstream targets" may not be the proper term for IFI6 and IFI27, and this concern needs to be clarified in both Results and Discussion sections.

19. Conceptual issue #2: In Fig. 4A, does RNA-seq analysis reveal any known ATF3 target genes in CAL27 cells? What are the known functions of IFI6 and IFI27, as well as their mechanisms of action, in normal development and/or disease pathogenesis? These questions should be carefully addressed in the manuscript, to provide the biological rationale for investigation of the ATF3-IFI6/IFI27 signaling cascade.

20. Technical issue: All *in vitro* validation studies solely rely on two cell lines, CAL27 and SCC-9. Another pair of $ATF3^{HIGH}$ and $ATF3^{LOW}$ cell lines need to be recruited and subjected to cell growth and migration assays, as well as RT-qPCR analysis of IFI6 and IFI27 gene expression.
