# Supplementary material for: ATF3 downmodulates its new targets IFI6 and IFI27 to suppress the growth and migration of tongue squamous cell carcinoma cells
Source: PLoS Genet. 2021 Feb 4;17(2):e1009283. doi: 10.1371/journal.pgen.1009283 (PMC7888615; doi:10.1371/journal.pgen.1009283)
Supplement: Attachment — Submitted filename: rebuttal letter - Plos Genetics-2020 10 30.docx [file pgen.1009283.s018.docx]

**RESPONSES to REVIEWERS’ COMMENTS**

**Reviewer #1:** The study by Lin Xu and colleagues discovered a tumor suppressor role for activating transcription factor 3 ATF3 in tongue squamous cell carcinomas TSCCs through negative regulation of IFI6 and IFI27. This finding provides a potentially important angle to understand the TSCC etiology. A thorough understanding of ATF3 alteration and its downstream signaling pathways may pave the way for inventing new therapeutics against TSCCs. On a broad

perspective, ATF3 regulation of IFI6 and IFI27 signaling could act as a fundamental mechanism conserved across diverse cancer forms and/or involved in normal development.

The authors have provided extensive molecular and cellular evidence, successfully connecting ATF3 function to the TSCC biology. Despite the strengths mentioned above, more experimental studies and conceptual clarification are required for general audience to efficiently and accurately digest the current manuscript.

**RESPONSE:** We thank the Reviewer for appreciating the novelty and potential impact of our study. We are grateful for such valuable comments and suggestions to improve our manuscript, which we feel has been significantly clarified and strengthened as a result. We have performed many new experiments as suggested and revised text is marked with underlining. Our point-by-point responses follow:

1. Page 5: The authors state that “a potential redistribution of ATF3 from nuclei to the cytoplasm”. This may not be true, since TSCC progression could theoretically prevent cytoplasmic ATF3 shuttling into the nucleus. The authors should revise the sentence – for example, ATF3 redistribution between nucleus and cytoplasm.

**RESPONSE:** We agree with this suggestion and we modified the text accordingly.

2. Suppl. Fig. 1: Is there any clinical staging difference between the five OSCC cell lines – highly, moderately, or poorly differentiated? Is there any scientific reason for using CAL27 and SCC-9 cells in this study?

**RESPONSE:** Thanks for these important questions. We carefully checked the datasheets of all TSCC cells purchased from the ATCC, as well as previous publications, but unfortunately we didn’t find any clear link between the TSCC cell lines with stages of clinical TSCC tumors. According to our findings, we assumed that TSCCs with a relatively low expression of ATF3 should be poorly differentiated tumors. The reason to choose CAL 27 and SCC-9 for our study was based on their expression levels of ATF3. CAL 27 cells express a relatively high level of ATF3 and were used for investigations of ATF3 function by deletion (loss of function) while SCC-9 cells express a relatively low level of ATF3 and were used for overexpression of ATF3 (gain of function). The study of TSCC cells with different expression levels of ATF3 will help us understand the detailed functional role of ATF3 in TSCC cells.

3. Fig. 1C: Please add the individual images for ATF3 staining only.

**RESPONSE:** Thanks for this suggestion and we provided the individual images in the revised Figure (S3 Fig)

4. Fig. 1E: Immunofluorescence quantification alone may not be enough to accurately determine the difference of nuclear/cytoplasmic ATF3 expression between the three cell lines. These observations should be confirmed by subcellular fractionation assay (cytoplasm/nucleoplasm/ chromatin), followed by western blot analysis.

**RESPONSE:** Thanks for this suggestion. We checked the ATF3 level in the cytoplasmic and nuclear fractions by western-blot analysis, which confirmed the staining results. These new data are shown in Fig 1F in the revised manuscript.

5. Fig. 1F: Please change the Y-axis values to a Log scale, to increase the readability.

**RESPONSE:** Thanks for this suggestion, and we changed it as suggested. We agree that the results look much clearer than in the previous Figure (now in Fig 1G).

6. Page 6: Please revise the conclusion paragraph, as no results directly support “the function of ATF3 is downregulated”, nor “disrupted localization of ATF3 from the nucleus to the cytoplasm”.

**RESPONSE:** We agree with this comment, and we revised the statement accordingly in the revision.

7. Page 6 (the third from last line): Please change (Fig. 1E, F) to (Fig.1E), since Fig. 1F represents clinical data, rather than cell culture work.

**RESPONSE:** Thanks for noticing this and we corrected the text accordingly, it should be “S2 Fig” in the revised manuscript.

8. Fig. 2F: Please repeat western blots in triplicate and perform relative quantification of both p-AKT/AKT and p-ERK/ERK in a bar chart (as illustrated in Fig. 3G).

**RESPONSE:** Thanks for this suggestion. We had forgotten to put the quantification data in the original version, and we have added it to the revised manuscript in Fig 3G.

9. Page 7: The authors state that “loss of ATF3 function promotes the malignant progression of oral SCCs”. But, this seems to be an overstatement in the absence of direct evidence from transgenic animal studies tracing the steps of OSCC development in vivo.

**RESPONSE:** Thanks and we agree with this comment. We revised that statement accordingly in the revised manuscript.

10. Page 7 (the ninth from last line): Please change (Fig. 1F) to (Fig.1E).

**RESPONSE:** Thanks for noticing this and we changed it accordingly in the revision.

11. Fig. 3B: Please replace the figure panel with color images (as shown in Fig. 2B).

**RESPONSE:** Thanks for recommending this and we replaced Fig 3B with color images accordingly.

12. Fig. 4B: Please add the SG2 data to RT-qPCR validation.

**RESPONSE:** Thanks for this suggestion. We added the SG2 data accordingly, which are now shown in Fig 4C of the revised manuscript.

13. Fig. 4D: How many patient samples were subjected to IHC analysis? Please provide IHC scores for IFI6 and IFI27 in normal vs. TSCC specimens.

**RESPONSE:** Thanks for this comment. Six patient samples were stained for IFI6, and we added that information to the Fig 4 legend. As we mentioned in the text, the upregulation of IFI27 in oral tumors has been previously reported and therefore we didn’t repeat this result in this study.

14. Fig. 4E: Please change the Y-axis values to a Log scale.

**RESPONSE:** Thanks for the suggestion, and we changed the Y-axis values accordingly in the revised Figure.

15. Figs. 5B and 5D: Which time point (day) was selected for RT-qPCR validation of knockdown/overexpression efficiency? This information should be added to the figure legend.

**RESPONSE:** Thanks for asking about this. Both were 48 h after transfection or infection and we added this information to the Fig 5 legend accordingly.

16. Fig. 5: There’s no description/discussion on Figs. 5E, 5F, 5G, 5H, and 5I.

**RESPONSE:** Thanks for noticing this and it was a mistake. We have added that information into the text accordingly in the revised manuscript.

17. Fig. 6: If possible, please change Fig. 6 to a color figure.

**RESPONSE:** Thanks for this suggestion and new Fig 6 is now a color figure in the revision, and the original mouse images were moved to S12 Fig and S13 Fig with color in the revision.

18. Conceptual issue #1: It’s unclear if the IFI6 and IFI27 genes are two direct targets of ATF3 (or its downstream effectors)? If yes, then ChIP-qPCR should be applied to prove ATF3 binding to the IFI6 and IFI27 promoters. If no, then “ATF3 downstream targets” may not be the proper term for IFI6 and IFI27, and this concern needs to be clarified in both Results and Discussion sections.

**RESPONSE:** Thank for this valuable comment and suggestion. We performed ChIP-assays to prove that ATF3 can directly bind the promoter regions of the IFI6 and IFI27 genes, and those data are now shown in Fig 4E and in S9 Fig in the revised manuscript.

19. Conceptual issue #2: In Fig. 4A, does RNA-seq analysis reveal any known ATF3 target genes in CAL27 cells? What are the known functions of IFI6 and IFI27, as well as their mechanisms of action, in normal development and/or disease pathogenesis? These questions should be carefully addressed in the manuscript, to provide the biological rationale for investigation of the ATF3-IFI6/IFI27 signaling cascade.

**RESPONSE:** Thanks for these constructive comments. According to your suggestion, we performed Gene Ontology (GO) enrichment analysis of pathways induced by the deletion of ATF3 in CAL 27 cells, and found that “Response to both Type I interferon (alpha, beta) and type II (gamma) interferon” pathways appeared in the Top 15 list of upregulated pathways induced by the deletion of ATF3. Further, gene cluster analysis revealed the increased expression of many cytokine genes, which contain known ATF3 targets such as IL-6 and IL-1b. Importantly, the interferon alpha induced genes IFI6 and IFI27 were in the list of Top genes most upregulated by ATF3 deletion. IFI6, has been shown to play anti-apoptosis role in gastric cancer cells, breast cancer cells, human myeloma cells and vascular endothelia cells mainly through inhibiting activation of caspase 3 and suppressing proapoptotic gene BCL-2 expression (Tahara et al., 2005; Cheriyath et al., 2007, 2012; Qi et al. 2015), and anti-apoptosis plays a crucial role for tumor development. Especially, IFI6 was recently reported to promote metastatic potential of breast cancer cells through mitochondrial ROS (mtROS) (Cheriyath et al., 2018,). IFI27 has been reported to be involved in different biological processes including the regulation of cellular apoptosis; modulation of the transcriptional activity of nuclear receptors NR4A1, NR4A2 and NR4A3; involvement in the ubiquitin-mediated proteasomal degradation pathway and playing antivirus functions ([Papac-Milicevic](https://pubmed.ncbi.nlm.nih.gov/?sort=pubdate&term=Papac-Milicevic+N&cauthor_id=22427340) et al., 2012; Xue et al., 2016; Rosebeck et al., 2008; Gytz et al., 2017; Liu et al., 2014). IFI27 has been shown to inhibit Heptatis C virus replication through activation of the JAK/STAT signaling pathway (Chen et al., 2017), which has been reported to be interact with AKT and MAPK/ERK pathways and to be associated with cancer development. However, IFI6 and IFI27 have not been previously reported to be targets of ATF3, and their roles in TSCC cells have not been studied. Therefore, it was very interesting for us to explore their functions in TSCCs in this study. We added this information into the revised manuscript.

20. Technical issue: All in vitro validation studies solely rely on two cell lines, CAL27 and SCC-9. Another pair of ATF3HIGH and ATF3LOW cell lines need to be recruited and subjected to cell growth and migration assays, as well as RT-qPCR analysis of IFI6 and IFI27 gene expression.

**RESPONSE**: According to this suggestion, two other cell lines (SCC-25 and SCC-4) were used for the analysis for cell growth and migration following the deletion or overexpression of ATF3, which is now shown in S7 Fig and S8 Fig.

**Reviewer #2:** The manuscript reports that ATF3 expression and nuclear localization negatively correlates with TSSC malignancy in vivo and growth and invasion in vitro. The low levels of ATF3 lead to upregulation of IFI6 and IFI27, which execute growth and migration of TSCCs. The manuscript reports the first association of ATF3 with IFI6 and IFI27. However, the study is preliminary and requires more work to become a strong paper.

(1) The ATF3 levels in cytoplasmic and nuclei in tumor tissues and cultured TSCCs were not biochemically tested (fractionation), (2) the growth differences of the cultured TSCCs with and without ATF3 is marginal, (3) the Crispr-mediated deletion of ATF3 is not confirmed by sequence analyses, etc., etc. (4) Moreover, it is unclear how ATF3 down regulates the IFI6/27,

(5) whether the IFI6/27 is dominant over the induction by the interferon produced upon white blood cell infiltration into the tumor tissues, and (6) how IFI6/27 promotes growth, migration and invasion of TSCCs.

**RESPONSE:** We thank the Reviewer for such constructive comments, and we completely agree with the concerns raised by the Reviewer. We addressed those comments point by point as follows:

(1) ATF3 levels in the nuclear and cytoplasmic fractions were analyzed in the revision and the results supported the staining data;

(2) We agree that the change of TSCC cell growth by ATF3 was not dramatic in *in vitro* cultures, but the difference when compared with the corresponding controls was statistically significant, which was also observed and repeated in all four TSCC cell lines. Importantly, we found that the inhibition of TSCC proliferation was much more severe in the *in vivo* tumor model, shown in revised Fig. 6. Taken together, these results suggest that there is potentially an unknown feedback signaling pathway activated by the deletion of ATF3 *in vitro*;

(3) We totally agree with the Reviewer’s comment that sequence analysis to confirm the CRISPR mediated cleavage is important, however it is more crucial to directly show the deletion of ATF3 protein by western-blot analysis and to observe the biological consequences of the loss of ATF3. We designed two gRNAs to specifically target ATF3: one gRNA (SG1) targets the end region of the ATF3 exon right before the 3’-UTR, which is expected to produce a truncated form of ATF3; the other gRNA (SG2) targets the end of the 5’-UTR of the ATF3 gene, which is expected to fully delete ATF3 in the CRISPR-CAS9 system. Western-blot analysis confirmed those expectations. Importantly, the same vector used for the CRISPR-mediated deletion of ATF3 was also used for human primary dermal fibroblasts in our recent publication (Zu et al., 2020). In agreement with that previous publication, our present study showed the same results and we think that the CRISPR-mediated deletion of ATF3 by SG1 results in a truncated form of ATF3, likely a non-functional form that produced all phenotypes similar to the SG2 mediated deletion (an efficient deletion revealed by western blot) both *in vitro* and *in vivo*. In addition, we used an siRNA to knockdown ATF3 and that knockdown of ATF3 further verified the increased growth of TSCCs elicited by SG1 and SG2 ATF3. Taking these data together, we are confident that the CRISPR mediated cleavage of ATF3 resulting in the loss of function is efficient.

(4) We found that IFI6 and IFI27 are potential targets of ATF3 by analyzing previously published ATF3 CHIP data, and our own CHIP assays indeed showed that ATF3 directly binds the promoter regions of the IFI6 and IFI27 genes, suggesting that ATF3, a transcriptional suppressor, negatively controls IFI6/27 expression by binding its promoter region in TSCC cells.

(5) GO (Gene Ontology) analysis of the RNA-seq data was performed and is shown in Fig. 4A in the revision. The GO analysis revealed that upregulation of “Response to both type I and type II IFN” pathways is one of the Top pathways in ATF3 deleted cells. IFI6 and IFI27 were identified in the list of top genes of these IFN induced genes in ATF3 deleted cells. GO analysis also revealed pathways related to the migration of immune cells, so effects on immune cell infiltration are also a potential mechanism to affect TSCC growth. Both *in vivo* and *in vitro* data demonstrated that the control of TSCC growth, migration and differentiation by ATF3 depends on IFI6 and IFI27. So, taking these data together, we proposed that ATF3 plays a tumor suppression function in TSCCs mainly through the negative regulation of IFI6 and IFI27 expression, but of course we can not exclude that other cytokines are also involved.

(6) IFI6, has been shown to play anti-apoptotic role in gastric cancer cells, breast cancer cells, human myeloma cells and vascular endothelia cells mainly through inhibiting activation of caspase 3 and suppressing proapoptotic gene BCL-2 expression (Tahara et al., 2005; Cheriyath et al., 2007, 2012; Qi et al. 2015), and anti-apoptosis plays a crucial role for tumor development. Especially, IFI6 was recently reported to promote metastatic potential of breast cancer cells through mitochondrial ROS (mtROS) (Cheriyath et al., 2018,). IFI27 has been reported to be involved in different biological processes including the regulation of cellular apoptosis; modulation of the transcriptional activity of nuclear receptors NR4A1, NR4A2 and NR4A3; involvement in the ubiquitin-mediated proteasomal degradation pathway and playing antivirus functions ([Papac-Milicevic](https://pubmed.ncbi.nlm.nih.gov/?sort=pubdate&term=Papac-Milicevic+N&cauthor_id=22427340) et al., 2012; Xue et al., 2016; Rosebeck et al., 2008; Gytz et al., 2017; Liu et al., 2014). IFI27 has been shown to inhibit Heptatis C virus replication through activation of the JAK/STAT signaling pathway (Chen et al., 2017), which has been reported to be interact with AKT and MAPK/ERK pathways and to be associated with cancer development. Therefore we expect that IFI6 and IFI27 may control TSCC cell growth and migration through regulation of the JAK/STAT and anti-apoptotic pathways, which we will pursue in the near future.

**Reviewer #3:** The work by Wang et al investigates the mechanisms of transformation in head and neck squamous cell carcinomas, specifically arising in the tongue epithelia (TSCC). Nuclear ATF3 is lower in less differentiated TSSC samples and in a public database. ATF3 represses the growth and migration of two TSCC cell lines and comprehensive gene expression analysis in one of these cell line plus/minus ATF3 gene deletion identified two targets IFI6 and IFI27. Both are highly expressed in TSCCs and their silencing blunts the increased growth/proliferation induced by ATF3 deletion. According to an epistatic role for IFI6 and IFI27, their overexpression counteracted the inhibitory effects of ATF3 overexpression.
The controversial positive/negative role of ATF3 in SCC has been previously reported, thus the 1. novelty for the field is limited and the manuscript, in its present form,

2. does not provide new insights into the mechanism(s) upstream or downstream of this stress-induced transcriptional regulator. It is not clear if IFI6 and IFI27 are direct targets for ATF3 and, 3. more importantly, how IFI6 and IFI27 exert their effect(s).

4. How do the present findings relate to a pro-tumorigenic function of ATF3 in early steps of SCC development as previously reported by Wu et al. (Nature 2010)?

5. There is also the need to resolve some technical concerns regarding ATF3 nuclear localization in IHC, comparing normal oral KC vs TSSC and ChIP analyses of IFI6 and IFI27 promoters (with and without ATF3 silencing). Addressing these points would strengthen the enthusiasm for the manuscript.

**RESPONSE:** Thanks for these constructive comments. We address these comments point by point in the following:

(1) More and more evidence has indicated that ATF3 plays either a tumor promoting or a tumor suppressing function which depends on the different types of cells, which most likely is due to its downstream targets specific to the cell type. Therefore, it is important to identify the specific downstream targets of ATF3 in different cancers, which has not been studied in TSCCs. Here we are the first study to identify IFI6 and IFI27 as direct targets of ATF3 in TSCC cells. We believe that a thorough understanding of ATF3 alteration and its downstream signaling pathways provides a potential to develop new therapeutics for the treatment of tumors such as TSCC.

(2) CHIP assays were performed in the revision and showed that ATF3 can directly bind the promoter regions of IFI6 and IFI27. This present study mainly focuses on downstream targets of ATF3 in regulating TSCC cell growth and migration. It will be also interesting to pursue upstream regulators of ATF3 in TSCC development as the Reviewer suggests and we hope to study that in the future.

(3) IFI6, has been shown to play anti-apoptosis role in gastric cancer cells, breast cancer cells, human myeloma cells and vascular endothelia cells mainly through inhibiting activation of caspase 3 and suppressing proapoptotic gene BCL-2 expression (Tahara et al., 2005; Cheriyath et al., 2007, 2012; Qi et al. 2015), and anti-apoptosis plays a crucial role for tumor development. Especially, IFI6 was recently reported to promote metastatic potential of breast cancer cells through regulation of mitochondrial ROS (mtROS) (Cheriyath et al., 2018,). IFI27 has been reported to be involved in different biological processes including the regulation of cellular apoptosis; modulation of the transcriptional activity of nuclear receptors NR4A1, NR4A2 and NR4A3; involvement in the ubiquitin-mediated proteasomal degradation pathway and playing antivirus functions ([Papac-Milicevic](https://pubmed.ncbi.nlm.nih.gov/?sort=pubdate&term=Papac-Milicevic+N&cauthor_id=22427340) et al., 2012; Xue et al., 2016; Rosebeck et al., 2008; Gytz et al., 2017; Liu et al., 2014). IFI27 has been shown to inhibit Heptatis C virus replication through activation of the JAK/STAT signaling pathway (Chen et al., 2017), which has been reported to be interact with AKT and MAPK/ERK pathways and to be associated with cancer development. Therefore we expect that IFI6 and IFI27 may control TSCC cell growth and migration through regulation of the JAK/STAT and anti-apoptotic pathways, which we will pursue in the near future.

(4) As we mentioned above, the function of ATF3 is likely determined by its specific downstream targets in different cells. In skin keratinocytes, ATF3 could target the inhibition of p53 to promote tumorigenesis, however in TSCCs, ATF3 directly targets IFI6 and IFI27 to suppress tumor cell growth and migration.

(5) As suggested, we performed western-blot analysis of ATF3 level in nuclear and cytoplasmic fractions and ChIP assays. Detailed responses to these comments and suggestions are listed point-by-point below.

**Specific points**

• A western blot of nuclear vs cytosolic fractions in SCC cells vs oral keratinocytes is required to hypothesize a cytosolic relocalization of ATF3 during transformation.

**RESPONSE:** Thanks for this valuable suggestion. We performed western blot analysis of ATF3 expression levels in the cytoplasmic and nuclear fractions, which is now shown in Fig 1F in the revision, and those results supported the staining data.

• The DAPI nuclear signal in the immunofluorescence in figure 1C should be separated to permit the detection of ATF3 nuclear signal. A confocal image of a higher magnification is required. On this line, a z section should be shown as the "nuclear" ATF3 red signal might be the result of a stacked signal (eg from the top of the cell).

**RESPONSE:** Thanks for this suggestion, and we now show individual images of ATF3 and DAPI in S3 Fig. Together with the western blot analysis of ATF3 levels in the nuclear fraction, we think that these data should be sufficient to support the lower nuclear expression of ATF3 in TSCC cells compared to normal oral epidermal cells.

• The levels in IF show that ATF3 levels are not decreased in TSSC (while the mRNA is), and accordingly a strong cytosolic ATF3 signal is detectable, the mechanism overseeing this important novelty should be investigated.

**RESPONSE:** Thanks for the comment, but we didn’t fully agree this observation. According to both staining and western blot data (Fig 1C,F), not only is the nuclear level of ATF3 dramatically lower in TSCCs, especially in poorly differentiated TSCCs, but also the total level of ATF3 is reduced, which also fits with the TCGA data (mRNA level) as shown in Fig. 1G.

• In figure 2A the differences in cal27 cell proliferation after AFT3 deletion, measured with the formazan-forming dye, are slim at best and after day 3 do not increase further, although the cells do not have ATF3. The authors should explain this transient effect. Do the cell die?

**RESPONSE:** Thanks for this observation. In order to make sure that what we observed is correct, we repeated the same experiment shown in Fig 2A, and also used another cell line (SCC-25, shown in S7 Fig) to reproduce the results. Both experiments produced very similar results, which could be due to an unknown feedback signaling pathway activated by the deletion of ATF3 *in vitro* (inhibition of TSCC proliferation was much more severe in *in vivo* tumors shown in Fig 6) or a low level of ATF3 (not 100% deletion by the CRISPR system) still plays a role in blocking the growth of TSCC cells, such as cell cycle arrest or differentiation. We added an explanation of this in the revised text.

• In figure 2E the western blot of Cal27 cells that underwent CRISPR-mediated deletion of ATF3 (with one of the SG guides) shows a band slightly smaller than ATF3 (there are no MW to evaluate the size), but which is still recognized by ATF3 Abs. This is of great concern as it might the result of a recombination/partial deletion leading to a smaller (but functional?) peptide. ATF3 mRNA levels should be tested and the results complemented by the use of ATF3 shRNAs

**RESPONSE:** Thanks for these comments. We designed two gRNAs to specifically target ATF3: one gRNA (SG1) targets the end region of the ATF3 exon right before the 3’-UTR, which is expected to produce a truncated form of ATF3; the other gRNA (SG2) targets the end of the 5’-UTR of the ATF3 gene, which is expected to fully delete ATF3 in the CRISPR-CAS9 system. Importantly, the vector used for the CRISPR-mediated deletion of ATF3 was also used for human primary dermal fibroblasts in our recent publication (Frontiers in Oncology, 2020). In agreement with that previous publication, our present study showed the same result and we think that the CRISPR-mediated deletion of ATF3 by SG1 results in a truncated form of ATF3, likely a non-functional form that produced all phenotypes similar to the SG2 mediated deletion, an efficient deletion revealed by western blot *in vitro* and *in vivo*. In addition, we used an siRNA to knockdown ATF3 and that knockdown of ATF3 further verified the increased growth of TSCCs elicited by SG1 and SG2 ATF3. We added this information into the revised version.

• From the presented data it is not clear how Interferon-alfa inducible protein 6 and 27 would exert their function(s) towards cell proliferation, transformation etc. More importantly, it is not known if these transcripts are direct targets for ATF3 in TSCC.

**RESPONSE:** Thanks for this valuable comment. CHIP assays were performed and showed that ATF3 directly binds the promoter regions of IFI6 and IFI27, indicating that they are direct targets of ATF3. Our data showed that the effects induced by ATF3 in TSCC cells can be counteracted by the expression of IFI6 or IFI27 both in *in vitro* cell cultures and in an *in vivo* tumor model, which suggests that ATF3 plays a tumor suppressing function in TSCCs through the negative regulation of IFI6 and IFI27.

As mentioned above, both IFI6 and IFI27 have been shown to play important roles in the regulation of cellular apoptosis in different cell types. IFI27 has been shown to activate the JAK/STAT signaling pathways, which are associated with cancer development. Therefore we expect that IFI6 and IFI27 may control TSCC growth and migration through regulation of the JAK/STAT and apoptotic signaling pathways, which we will pursue in the near future.

**Reviewer #4:** In this report, Xu et al., has described interesting findings of the tumour suppressor role of ATF3 in oral squamous cell carcinoma with IFI6 and 27 as the prominent downstream targets. The study design is straightforward and the results showed are intriguing.

**RESPONSE:** We thank the Reviewer for the positive comments.

**Major points:**

• SCCO28 is a highly metastasis-like oral cancer cell line, how would the authors explain the high expression there?

**RESPONSE:** Thanks for this comment and that was a mistake; SCC-25 cells, not SCCO28 cells, were studied. We revised the manuscript to correct that terminology.

• The work is heavily relayed on 2D cell scratching assay, which only represent the migration potential of the cells, while at least cell differentiation has not been measured.

**RESPONSE:** Thanks for the valuable comment. We investigated the role of ATF3 in regulating TSCC cell differentiation *in vivo*, and indeed, the overexpression of ATF3 promoted TSCC cell differentiation, which is shown in Fig. 6 I-N.

• Figure 1. For human oral SCC, please include low magnification for overview of the clinical samples, and multiple samples for each category of the cancer. Please mark epithelial-mesenchymal junctions Panel B labelling is misaligned.

**RESPONSE:** Thanks for these constructive comments. We now provide representative images of three patient samples from each category including low magnification images for Fig 1B. And we also corrected the labeling.

• Figure 6. The analysis of tumourigenesis assay are missing, differentiation, proliferation and apoptosis etc. have to be analysed to complete the story. Also are there any metastasis signs? Please consider to remove the photos of mice. For panel B and E, scales are missing. For C and F, the individual sample has to be indicated on the plot.

**RESPONSE:** Thanks for these constructive comments. According to your suggestion, we performed IHC staining to analyze cell proliferation and differentiation in the xenografted tumors, and these data are now shown in Fig 6. We didn’t see any signs of metastasis by checking lung and liver tissues. And as suggested, we moved the mouse images to S12 Fig and S13 Fig, added the scale bars and replaced the graph with solid dots to indicate individual samples.

**Minor points:**

1. Full names for IFI etc. have to be indicated at the first instance.

**RESPONSE:** Thanks for noticing this and we defined all abbreviations at first use.

2. What do you mean "disrupted localization of ATF3 from the nucleus to cytoplasm"? shouldn't that be opposite?

**RESPONSE:** Thanks for this comment and sorry for the confusion. We revised the text accordingly.

3. You should acknowledge and cite the original provider for SCC4 and SCCO28.

**RESPONSE:** Thanks for this suggestion and we provided those details accordingly, and as noted above, SCCO28 should be SCC-25. Both SCC-4 and SCC-25 cells were from the ATCC.

4. What was the normal oral epithelial culture condition?

**RESPONSE:** We have now provided that information in the revised Materials and Methods.

5. For puromycin selection on cancer cells, are 48 enough? If yes do you have the control and proof?

**RESPONSE:** Thanks for these questions. That was a mistake and it was 6 days of selection, and we corrected that in the revised manuscript.

6. For antibody (IHC, IF and WB), there are many writing mistakes. Please indicate the antibody full name (such as HRP conjugated etc.) working concentration and company's full name.

**RESPONSE:** Thanks for this comment and we provided details of all antibodies (names, sources, etc) in the revised manuscript as suggested.

7. What is the siRNA concentration?

**RESPONSE:** The siRNA concentration was 20 nM, and we now provide that in the revised Materials and Methods.

8. 36B4 should be 36beta4.

**RESPONSE:** Thanks for noticing this and we corrected it accordingly.

9. Which sever has the RNA-seq result been deposited? Which access number?

**RESPONSE:** Thanks for asking us to provide this information, which has been added to the revised manuscript.

10. nude/nude mice are which genetic background?

**RESPONSE:** Thanks for this question. We have added the genetic background of those mice in the revised manuscript.

11. "P value" should be "p-value".

**RESPONSE:** Thanks for this comment and we corrected that accordingly.

12. Molecular weights for the WB bands should be indicated.

**RESPONSE:** Thanks for the comment. We provided the molecular weights in the corresponding Figures.
